# Hypoxia-Associated Prognostic Markers and Competing Endogenous RNA Co-Expression Networks in Breast Cancer

**DOI:** 10.3389/fonc.2020.579868

**Published:** 2020-12-02

**Authors:** Peng-Ju Gong, You-Cheng Shao, Si-Rui Huang, Yi-Fan Zeng, Xiao-Ning Yuan, Jing-Jing Xu, Wei-Nan Yin, Lei Wei, Jing-Wei Zhang

**Affiliations:** ^1^ Department of Breast and Thyroid Surgery, Zhongnan Hospital, Hubei Key Laboratory of Tumor Biological Behaviors, Hubei Cancer Clinical Study Center, Wuhan University, Wuhan, China; ^2^ Department of Pathology and Pathophysiology, Hubei Provincial Key Laboratory of Developmentally Originated Disease, School of Basic Medical Sciences, Wuhan University, Wuhan, China

**Keywords:** hypoxia, breast cancer, The Cancer Genome Atlas, ceRNA, prognosis

## Abstract

**Objective:**

Many primary tumors have insufficient supply of molecular oxygen, called hypoxia. Hypoxia is one of the leading characteristics of solid tumors resulting in a higher risk of local failure and distant metastasis. It is quite necessary to investigate the hypoxia associated molecular hallmarks in breast cancer.

**Materials and Methods:**

According to the published studies, we selected 13 hypoxia related gene expression signature to define the hypoxia status of breast cancer using ConsensusClusterPlus package based on the data from The Cancer Genome Atlas (TCGA). Subsequently, we characterized the infiltration of 24 immune cell types under different hypoxic conditions. Furthermore, the differentially expressed hypoxia associated microRNAs, mRNAs and related signaling pathways were analyzed and depicted. On this basis, a series of prognostic markers related to hypoxia were identified and ceRNA co-expression networks were constructed.

**Results:**

Two subgroups (cluster1 and cluster2) were identified and the 13 hypoxia related gene signature were all up-regulated in cluster1. Thus, we defined the cluster1 as “hypoxic subgroup” compared with cluster2. The infiltration of CD8+ T cell and CD4+ T cell were lower in cluster1 while the nTreg cell and iTreg cell were higher, indicating that there was immunosuppressive status in cluster1. We observed widespread hypoxia-associated dysregulation of microRNAs and mRNAs. Next, a risk signature for predicting prognosis of breast cancer patients was established based on 12 dysregulated hypoxia associated prognostic genes. Two microRNAs, hsa-miR-210-3p and hsa-miR-190b, with the most significant absolute logFC value were related to unfavorable and better prognosis, respectively. Several long non-coding RNAs were predicted to be microRNA targets and positively correlated with two selected mRNAs, CPEB2 and BCL11A. Predictions based on the LINC00899/PSMG3-AS1/PAXIP1-AS1- hsa-miR-210-3p-CPEB2 and SNHG16- hsa-miR-190b-BCL11A ceRNA regulation networks indicated that the two genes might act as tumor suppressor and oncogene, respectively.

**Conclusion:**

Hypoxia plays an important role in the initiation and progression of breast cancer. Our research provides potential mechanisms into molecular-level understanding of tumor hypoxia.

## Introduction

Statistics from the International Cancer Research Center show that breast cancer morbidity and mortality rank first and second among female tumors. Worldwide, the incidence of breast cancer is increasing year by year, and the age of onset is getting younger (1). Although China belongs to a region with relatively low incidence of breast cancer, the number of new cases of breast cancer has been increasing in recent years. In some large and medium-sized cities, it has risen to the top of the incidence of female malignant tumors, which seriously threatens the health and lives of women and brings a huge economic and health burden to society. With the advancement of treatment, the prognosis of breast cancer continues to improve, but breast cancer is still a dominant cause of death for women at present (2).

During the growth of malignant tumors, tumor cells grow faster than their blood vessels grow. The effective oxygen diffusion range of the capillaries around the tumor cells cannot meet the needs of rapid tumor growth, resulting in uneven supply of oxygen and nutrients in tumor tissue, thereby forming the hypoxic microenvironment (3, 4). Hypoxia is one of the leading characteristics of solid tumors including breast cancer, and plays an important role in the occurrence and development of cancers (5). Hypoxia in the local microenvironment can promote the formation of new blood vessels by inducing Hypoxia-inducible factor 1-alpha (HIF-1α) (6), Vascular endothelial growth factor (VEGF) (7), C-C Motif Chemokine Ligand 28 (CCL28) (8) and other cytokines, and regulate the expression of the signal cascade and downstream related genes, thereby promoting the proliferation and invasion of tumor cells (9). For example, it has been demonstrated that the expression level of HIF-1α in breast cancer and other tumor tissues is significantly higher than that in adjacent tissues, and its increase is positively correlated with the incidence of breast cancer metastasis and mortality (10). Therefore, exploring the exact or related mechanism of hypoxia in tumorigenesis and development is expected to provide new targets and indicators for the treatment and prognostic detection of breast cancer. However, due to variations of oxygen levels in different tissues, it is still difficult to determine the hypoxia status in tumors. Current research shows that under hypoxic conditions, tumor cells can adapt to the microenvironment on which they grow by changing the expression of enriched endogenous genes, and these gene expression signatures can reflect hypoxia status (11–13).

The discovery of a series of non-coding RNAs in recent years, including long non-coding RNA (lncRNA), microRNA (miRNA), Circular RNA (circRNA), etc., has uncovered new ways of regulating gene expression in eukaryotes. Non-coding RNAs are involved in a variety of pathological processes, especially in the occurrence and development of tumors (14). A miRNA is a small non-coding RNA molecule containing about 22 nucleotides, and can inhibit the expression of target genes by completely or incompletely binding the 3’UTR region of the target genes’ mRNA. A lncRNA is a type of non-coding over 200 nucleotides in length with no protein coding potential, and can positively or negatively regulate gene expression through various mechanisms. The competitive endogenous RNAs (ceRNAs) hypothesis is one theory that explains the mechanism of lncRNA and miRNA. The theory proposes that lncRNA competes with miRNA for binding and covers the miRNA response element, thereby mitigating the inhibitory effect of miRNA on target mRNAs (15). Besides, lncRNA acts as a molecular sponge of miRNA to inhibit the expression of miRNA (16). The “lncRNA-miRNA-mRNA” network has been confirmed in many human cancers (17).

In this study, we used 13 hypoxia-related gene expression signature to characterize the different hypoxia states of breast cancer samples in The Cancer Genome Atlas (TCGA), and depicted the infiltration of 24 immune cell types in breast cancer tissues under different hypoxic conditions. Furthermore, the differentially expressed hypoxia associated miRNAs, mRNAs and related signaling pathways were analyzed and investigated. On this basis, a series of prognostic markers related to hypoxia were screened and a ceRNA co-expression network in breast cancer was constructed. These results have the potential to further improve the regulatory mechanisms under hypoxia in breast cancer.

## Materials and Methods

### Study Cohort

The Cancer Genome Atlas (TCGA) breast invasive carcinoma (BRCA) gene expression profile and miRNA mature strand expression RNAseq illuminaHiseq data were retrieved from UCSC Xena (18, 19), as in log2(X+1) transformed RSEM normalize count. The gene expression data included 1,104 tumor samples and 114 normal samples as control. Exchanging the Accession number to the ID of miRNA was performed by miRbase database (20) and miRBaseVersions.db R package. Besides, the phenotype of BRCA samples was also gained from UCSC Xena. Among them, 703 samples have complete clinical and pathological data. This study complied with the publication guidelines of TCGA, and ethics approval and informed consent were not required. For tested cohort, mRNA expression Z scores data and clinical data of 1,356 breast cancer tumor samples were obtained from the Molecular Taxonomy of Breast Cancer International Consortium (METABRIC) dataset (21) on cBioPortal online database (22, 23).

### Classification of Hypoxia Status

According to the studies published, we selected 13 hypoxia related gene expression signature for our analysis: ADM, TUBB6, MRPS17, CDKN3, TPI1, ALDOA, MIF, PGAM1, LDHA, P4HA1, SLC2A1, NDRG1, and VEGFA, which have been shown to perform the hypoxia status (12, 24). Cellular pathways of 13 hypoxia related gene signature were shown in **Table S1** for details. Spearman’s rank correlation was performed to assess the correlation among these gene by corrplot package, and the PPI network was built using the STRING database (25). Furthermore, two different hypoxia status groups (cluster1 and cluster2) among 1104 TCGA BRCA tumor samples were selected by using ConsensusClusterPlus package with 50 iterations, resample rate of 0.8. Principal component analysis (PCA) was analyzed and visualized by limma package and ggplot2 package. The differential expression of these genes between tumor samples and normal samples, between cluster1 and cluster2 were analyzed by limma package with a cut-0ff P <0.05, then visualized by pheatmap and vioplot.

### Immune Cells Infiltration Analysis

The data of infiltration score and 24 immune cell types including 18 T-cell subtypes and 6 other immune cells: B cell, NK cell, Monocyte cell, Macrophage cell, Neutrophil cell and DC cell in TCGA BRCA was estimated and acquired from the ImmuCellAI database (26). Then, the relationship of these cells and hypoxia status was analyzed by limma package with a cut-0ff P <0.05. The prognostic values of CD4+ T cell, CD8+ T cell, iTreg cell and nTreg cell were calculated via the Kaplan-Meier analysis and Logrank-P test by Graphpad 8.0, and X-tile software (27) was used to determine the optimal cut-off point for the prognostic value of these four types of cells.

### Differentially Expressed Genes (DEGs) Analysis

Limma package was utilized to identify differentially expressed genes between cluster1 and cluster2 and adjusted P value < 0.05 and |logFC|≥1 were considered to have a significant difference. Then, the PPI network of DEGs was constructed via STRING database (25), and the crucial sub-network was selected by the MCODE APP on Cytoscape 3.7.2 (28) according to the rules as follow: degree cut-off = 10, node score cut-off = 0.2, Max depth = 100, and k-Score = 2. Further, the pathway enrichment of the sub-network was analyzed by the ClueGo APP on Cytoscape 3.7.2, and the Gene Ontology (GO) enrichment analysis, including: biological processes (BP), cellular components (CC) and molecular functions (MF), was performed via STRING analysis category.

### Identification of Hypoxia Associated Prognostic Markers Among DEGs

The prognostic related genes were identified by univariate Cox regression analysis. After that, LASSO Cox regression was employed to select the powerful independent prognostic markers with P<0.05 for OS in BRCA. The risk score (RS) was calculated by the following formula:

$$RS=\sum_{i=1}^{n}Coef(i)X(i)$$

Where n represents the gene number in the module, Coef (i) is the coefficient of each gene; X(i) means the mRNA expression level of each gene. When Coef (i) is less than 0, it means that the corresponding gene plays a protective effect on the patient. When Coef (i) is greater than 0, the gene represents the opposite trend for survival. The TCGA BRCA tumor samples were divided into high rick and low risk groups by the cut-off of the median RS. Then, the prognostic value of RS in two groups was analyzed by Kaplan Meier method, and sensitivity and specificity assessments were estimated using the receiver operating characteristic (ROC) curves. Additionally, the relationship of RS and clinical parameters were also evaluated. This risk signature was validated by using Gene expression data and clinical data of 1356 breast cancer patients from METABRIC (21). Importantly, patients in Metabric dataset were divided into high risk and low risk groups by the optimal cut-off point of risk score which was obtained by X-tile software (27).

### Differentially Expressed MicroRNAs Analysis

The differentially expressed microRNAs between cluster1 and cluster2 were analyzed by limma package with adjusted P value < 0.05 and |logFC|≥1. The prognostic values of differentially expressed microRNAs in breast cancer were assessed by the online tools and database, Kaplan-Meier Plotter (29), and patient samples were divided into two groups by the best cut-off value by the tool automatically and calculated via the Kaplan-Meier analysis and Logrank-P test for the 120 months’ OS. We selected one microRNA with the highest |logFC| value in each of the up-regulated and down-regulated microRNAs for the next analysis. The targets genes of candidate microRNAs, hsa-miR-210-3p and hsa-miR-190b, were identified via the mirDIP database (30, 31), an integrative Database of Human microRNA Target Predictions, with the predict score as “very high”. Further, the GO enrichment analysis of the target genes was performed by STRING database, and the pathway enrichment was analyzed by the ClueGo APP on Cytoscape 3.7.2.

### Identify Genes Regulated by Candidate MicroRNAs Under Hypoxia

Venn diagrams were used to select the intersection of hsa-miR-210-3p targets and down-regulated genes, as well as hsa-miR-190b targets and up-regulated genes. The correlations between the selected genes and microRNAs were calculated via the Starbase database (32) based on the date from TCGA BRCA, and the prognostic values of selected candidate genes were analyzed by Kaplan-Meier Plotter database (29).

### Identify Target Long Non-Coding RNAs (lncRNAs) of Candidate MicroRNAs

StarBase database was employed to predict the target lncRNAs of candidate microRNAs, hsa-miR-210-3p and hsa-miR-190b, and the microRNAs-lncRNAs network were constructed via Cytoscape 3.7.2. Further, in order to acquire the confidence target lncRNAs in BRCA, lncRNAs were selected based on a negative relationship with candidate microRNAs (P value < 0.05, correlation coefficient <−0.1) and a positive relationship with target genes (P value < 0.05, correlation coefficient > 0.1) in the data collected from TCGA BRCA using StarBase database (32), and also chosen based on the prognostic values performed by the Kaplan-Meier Plotter (29).

### Construction of the Competitive Endogenous RNA (ceRNA) Network and Related PPI Network

STRING database was used to predict protein-protein interactions of candidate genes, and PPI network has been extended until the proteins in our analysis were connected to each other (25). Candidate microRNAs and lncRNAs were then added to the network.

### Statistical Analysis

Most of the statistical analysis were performed by online bioinformatic databases and tools as mentioned. Wilcox Test was employed to compare mRNA expression, infiltration score of immune cells and risk sore when comparing two sets of data. Chi-square test is used to compare clinical and pathological parameters and other categorical variables. Differentially expressed microRNAs and mRNAs were calculated by limma R package. The Kaplan-Meier curve and Log-rank P test, Univariable COX and LASSO Cox regression were used to analyze the survival outcomes. ROC curve was utilized to assess diagnostic effect. P-values < 0.05 were considered statistically significant. The visualization of the data was done by R 3.6.3, Excel 2019, Graphpad 8.0 and Cytoscape 3.7.2.

## Results

### Consensus Clustering Identified Two Clusters of BRCA With Different Hypoxia Status

**Figure 1:**
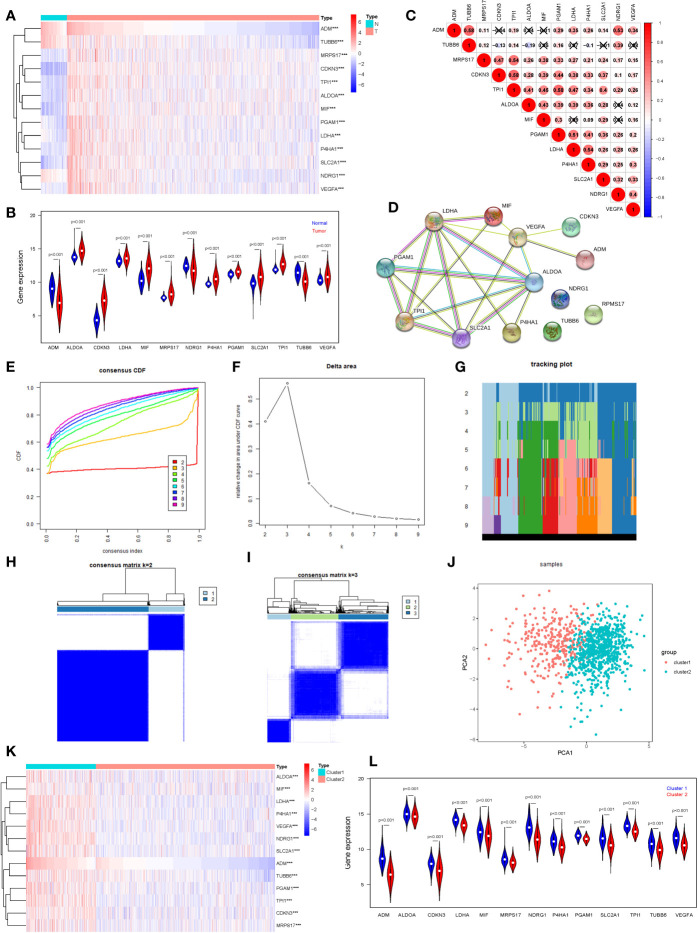
Consensus Clustering identified two clusters of BRCA with different hypoxia status. **(A)** The heatmap of the 13 hypoxia related gene expression signature in TCGA BRCA tumor and normal samples (Wilcox Test). **(B)** The expression of the 13 hypoxia related gene expression signature in TCGA BRCA tumor and normal samples (Wilcox Test). **(C)** Spearman correlation analysis of the 13 hypoxia related gene expression signature. **(D)** The PPI network of the 13 hypoxia related gene expression signature. **(E)** The CDF value of consensus index. **(F)** Relative change in area under CDF curve for k = 2–9. **(G)** The tracking plot for k = 2 to k = 9. **(H, I)** Consensus matrix for k=2 and k=3. **(J)** Principal component analysis of the total RNA expression profile. **(K)** The heatmap of the 13 hypoxia related gene expression signature in cluster1 and cluster2 (Wilcox Test). **(L)** The expression of the 13 hypoxia related gene expression signature in cluster1 and cluster2 (Wilcox Test). ***P < 0.001.

We selected 13 hypoxia related gene expression signature for our analysis: ADM, TUBB6, MRPS17, CDKN3, TPI1, ALDOA, MIF, PGAM1, LDHA, P4HA1, SLC2A1, NDRG1, and VEGFA. These genes were defined based on hypoxia-related gene function and were highly enriched for hypoxia-regulated pathways (11, 12), and their cellular pathways and functions were shown in **Table S1** for details. In order to understand their roles in oncogenesis in breast cancer, we firstly explored expression levels of these signature in tumor samples (n=1,104) and normal samples (n=114). The results are displayed as heatmap and vioplot, suggesting that all of them are abnormally expressed in BRCA samples. More specific, ADM, NDRG1, and TUBB6 were down-regulated, while other 10 genes were up-regulated in tumor samples compared with normal control (**Figures 1A, B**). Next, we analyzed the interrelationships and correlations between the 13 genes. Almost all of them were significantly associated with each other (**Figure 1C**). Except NDRG1, RPMS17 and TUBB6, other 10 genes can be incorporated into one PPI network (**Figure 1D**).

In order to define hypoxia status of 1104 TCGA BRCA tumor samples, based on the expression similarity of the 13 hypoxia related gene signature, Consensus Clustering Method was use to to cluster the samples. In the CDF curve of consensus matrix, there is a flattest middle segment of CDF curve when K=2 (**Figures 1E, F** and **SUPPLEMENTARY METHODS**). Besides, we noticed that the interference between subgroups could be reduced to minimal when K=2 was selected for consensus clustering analysis (**Figures 1G–I**). Thus, two subgroups named cluster1 (n=310) and cluster2 (n=794) were identified. Then, PCA was used to compare the transcriptional profile between cluster1 and cluster2, suggesting that there was a significant distinction between the two subgroups (**Figure 1J**). To better understand the hypoxia status of the two subgroups, we explore the expression of the 13 hypoxia related genes between cluster1 and cluster2. The result demonstrated that all of these 13 genes were up-regulated in cluster1. So, we could define the cluster1 as “hypoxic subgroup” compared with cluster2 (**Figures 1K, L**). After excluding cases with incomplete clinical data of TCGA BRCA from UCSC Xena, 703 cases were included to analyzed the association between hypoxia status and clinicopathological characteristics through chi-square test. The results showed that the hypoxia status was significantly associated with ER status, PR status, Her2 status and PAM50 subtype. In more detail, there were more ER-, PR-, Her2+ and triple negative breast cancer patients in cluster1 than cluster2 (**Table 1**).

**Table 1:** Association between hypoxia status and clinicopathological characteristics in breast cancer patients.

characteristic	Total	Cluster1 (199)		Cluster2 (504)		P Value*
		No. of patients	%	No. of patients	%	
ER Status						
Negative	161	104	52.26	57	11.31	<0.001
Positive	542	95	47.74	447	88.69	
PR Status						
Negative	227	122	61.31	105	20.83	<0.001
Positive	476	77	38.69	399	79.17	
HER2 Status						
Negative	597	159	79.90	438	86.90	0.019
Positive	106	40	20.10	66	13.10	
PAM50 Subtype						
Her2	53	33	16.58	20	3.97	<0.001
Luminal A	336	29	14.57	307	60.91	
Luminal B	160	40	20.10	120	23.81	
Basal/Normal	137	92	46.23	45	8.93	
T Stage						
T1-T2	602	171	85.93	431	85.52	0.888
T3-T4	101	28	14.07	73	14.48	
N Stage						
N0-N1	579	162	81.41	417	82.74	0.677
N2-N3	124	37	18.59	87	17.26	
M Stage						
M0	648	179	89.95	469	93.06	0.440
M1	13	5	2.51	8	1.59	
MX	42	15	7.54	27	5.36	
Stage						
Stage I-II	532	150	75.38	382	75.79	0.107
Stage III-IV	171	49	24.62	122	24.21	

*Chi-square Test.

### Immune Cells Infiltration of Different Hypoxia Status in BRCA

**Figure 2:**
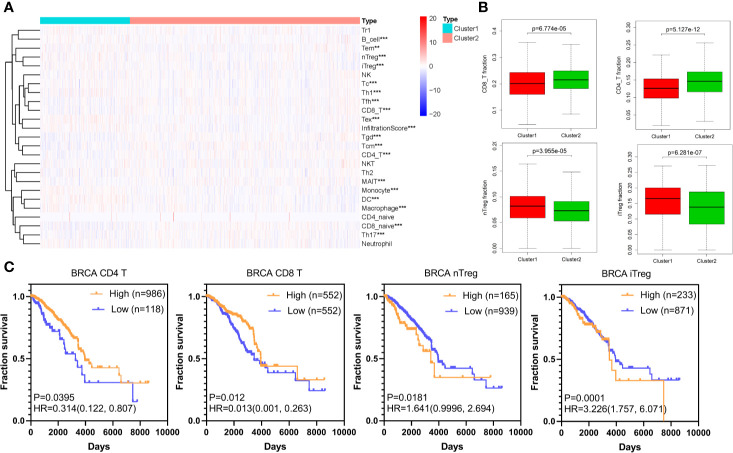
Immune cells infiltration of different hypoxia status in BRCA. **(A)** The heatmap of infiltration score and 24 immune cell types in cluster1 and cluster2 (Wilcox Test). **(B)** The infiltration of CD8+ T cell, CD4+ T cell, nTreg cell, and iTreg cell in cluster1 and cluster2 (Wilcox Test). **(C)** The overall survival curves of CD8+ T cell, CD4+ T cell, nTreg cell, and iTreg cell in TCGA BRCA. Log-rank *p* < 0.05 was considered statistical significance. HR > 1, cells infiltration was negatively associated with OS, while HR < 1, cells infiltration was positively associated with OS. **P < 0.01, and ***P < 0.001.

According to published articles, hypoxia is an important feature of tumors, which can regulate the immune response in tumors. Hypoxia induces tumor cells to produce multiple mechanisms by activating downstream signaling pathways to escape recognition and attack by the immune system (33). We analyzed 24 immune cell types including 18 T-cell subtypes and 6 other immune cells: B cell, NK cell, Monocyte cell, Macrophage cell, Neutrophil cell and DC cell in BRCA based on the ImmuCellAI database. The differences of these cells between cluster1 and cluster2 are shown in **Figure 2A** and **Figure S1**. Importantly, the CD8+ T cell and CD4+ T cell, immune cells that mainly recognize and kill tumor cells, were lower in cluster1 compared with cluster2, while the nTreg cell and iTreg cell were higher in cluster1 (**Figure 2B**). This result indicated that there was immunosuppressive state in cluster1. Further, the Kaplan-Meier analysis demonstrated that low CD8+ T cell and CD4+ T cell infiltration in TCGA BRCA tumor samples could predict a poor prognosis (P<0.05). Besides, there was a certain trend between poor survival prognosis and high iTreg cell and nTreg cell infiltration (P<0.05) (**Figure 2C**).

### Identification of Differentially Expressed Genes (DEGs) and Enrichment Analysis

**Figure 3:**
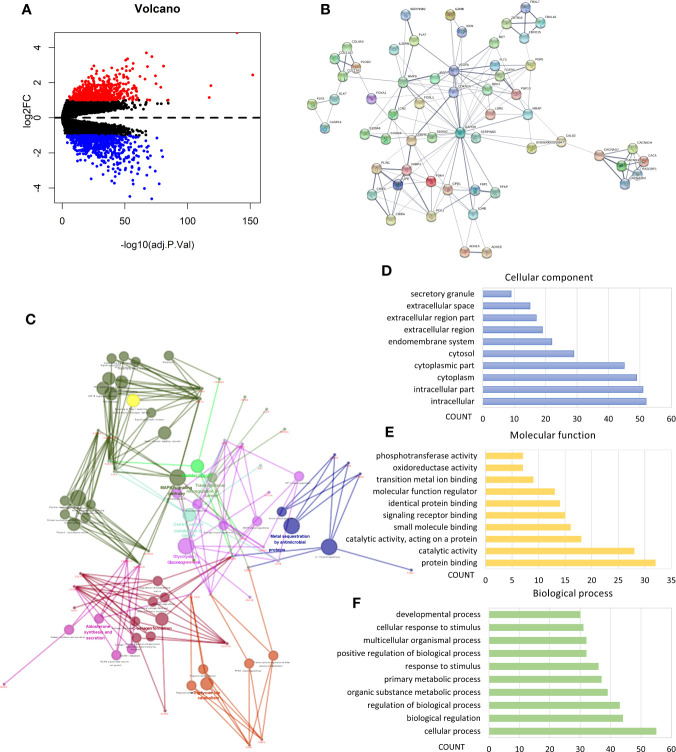
Identification of differentially expressed genes (DEGs) and Enrichment analysis. **(A)** Volcano plot for DEGs in cluster1 and cluster2. Red and blue dots represent up-regulated and down-regulated DEGs in cluster1 relative to cluster2, respectively (*P* < 0.05, |logFC| >1). **(B)** PPI network of a crucial sub-network with 58 nodes and 160 edges among DEGs. **(C)** Pathway enrichment analysis of the DEGs in the sub-network. **(D–F)** The top 10 items of GO analysis: biological processes, molecular functions and cellular components of the DEGs in the sub-network.

We performed analysis by the gene expression profiles of BRCA to identify hypoxia associated differentially expressed genes. A total of 1,225 differentially expressed genes were selected; 566 were up-regulated and 659 were down-regulated in cluster1 relative to cluster2 (**Figure 3A**). Considering that the number of differential genes was too large, it was difficult to accurately make GO and pathway enrichment analysis directly. First off, the PPI network of DEGs was constructed on STRING database, and then a crucial sub-network with 58 nodes and 160 edges was selected by the MCODE APP on Cytoscape 3.7.2 (**Figure 3B**). The top 10 items of each GO analysis: biological processes, molecular functions and cellular components, were shown as Bar graphs in **Figures 3D–F** (more details were depicted in **Table S2**). The pathway enrichment analysis showed that the DEGs in the sub-network linked to hypoxia and tumor related pathways: HIF-1 signaling pathway, Transcriptional misregulation in cancer, Bladder cancer, Central carbon metabolism in cancer, Glycolysis/Gluconeogenesis, AMPK signaling pathway, etc. (**Figure 3C**, **Table S2**). These results mean that these DEGs may play a role in promoting tumor progression through their function.

### Hypoxia Associated Prognostic Markers Among DEGs and a Risk Signature Established

**Figure 4:**
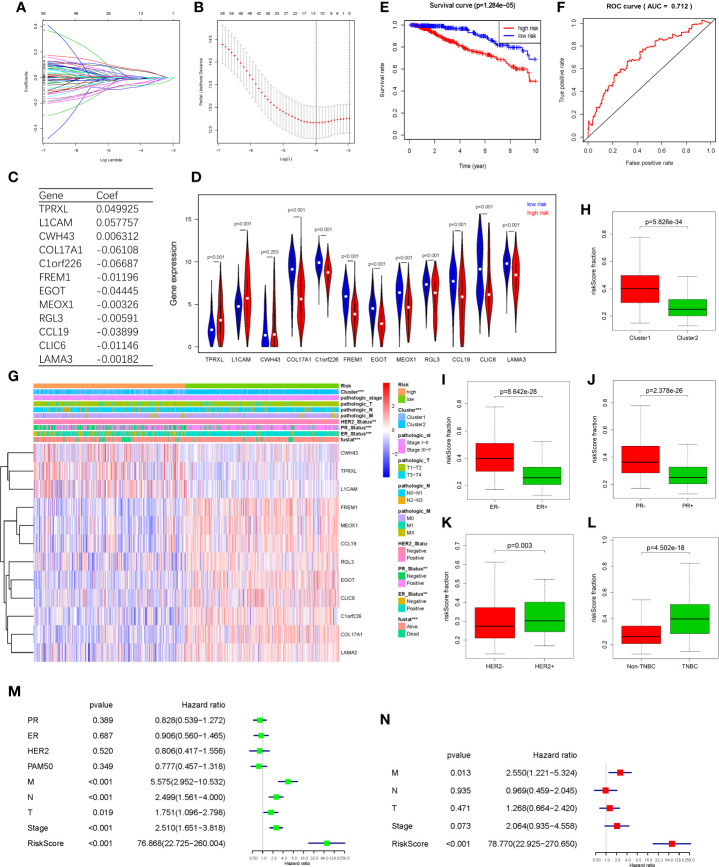
Hypoxia associated prognostic mRNA markers among DEGs and a risk signature. Established. **(A, B)** LASSO Cox regression was conducted to construct the most powerful prognostic markers. **(C)** The coefficients estimated by multivariate Cox regression via LASSO. **(D)** The expression of the 12 powerful prognostic markers in high-risk group and low-risk group (Wilcox Test). **(E)** Kaplan-Meier overall survival (OS) curves for patients in high- and low-risk group **(F)** ROC analysis and AUC value of the ROC curve for risk score. **(G)** The heatmap shows the expression of the 12 powerful prognostic markers in high-risk group and low-risk group (Chi-square Test). The distribution of clinicopathological characteristics was compared between the high-risk and low-risk groups. **(H–L)** The relationship between Rick score and ER, PR, HER2, TNBC (Wilcox Test). **(M)** Univariate Cox regression analysis of the associated between clinicopathological features (including risk score) and overall survival of patients. **(N)** Multivariate Cox regression analysis of the associated between clinicopathological features (including risk score) and overall survival of patients. **P < 0.01 and ***P < 0.001.

In order to find hypoxia associated prognostic makers among DEGs, univariate Cox regression analysis was performed based on the RNAseq illuminaHiseq data and overall survival (OS) data of 703 TCGA BRCA tumor samples. As a result, 15 up-regulated genes and 52 down-regulated genes were found significantly associated with OS (P<0.05) (**Table 2**). To identify the most powerful prognostic mRNA markers, the LASSO Cox regression analysis results (**Figures 4A, B**) demonstrated that 3 up-regulated and 9 down-regulated genes could be the powerful prognostic markers, and the coefficient of each gene were shown in **Figure 4C**. The related pathways and functions of these 12 genes were revealed in **Table S3**. Based on the 12 powerful prognostic markers, a risk signature was constructed, Then, the risk score was calculated based on the coefficient of each mark obtained from the LASSO analysis as follows: risk score = (0.049925* expression level of TPRXL) + (0.057757* expression level of L1CAM) + (0.006312* expression level of CWH43) + (−0.06108* expression level of COL17A1) + (−0.06687* expression level of C1orf226) + (−0.01196* expression level of FREM1) + (−0.04445 * expression level of EGOT) + (−0.00326* expression level of MEOX1) + (−0.00591* expression level of RGL3) + (−0.03899* expression level of CCL19) + (−0.01146* expression level of CLIC6) + (−0.00182* expression level of LAMA3). The TCGA BRCA tumor samples were divided into high-risk and low-risk groups according to the median risk score. The results of Kaplan-Meier analysis showed that the high-risk group had significantly worse prognosis compared with low-risk group (**Figure 4E**). In order to assess the sensitivity and specificity of the prediction, the AUC value was 0.712 obtained from Time-dependent ROC curve, suggesting well-prediction ability (**Figure 4F**).

**Table 2:** Prognosis-related DEGs in breast cancer.

Gene	HR[exp(coef)]	coef	95% CI lower	95% CI upper	P value*	log2FC	FDR**	Regulated
AKR1E2	1.129	0.121	0.002	0.241	0.047	1.433	8.57E-33	Up-Regulated
CASP14	1.066	0.064	0.003	0.125	0.040	3.483	2.45E-72	Up-Regulated
CWH43	1.088	0.084	0.004	0.165	0.040	1.321	3.58E-16	Up-Regulated
DPYSL5	1.083	0.079	0.001	0.157	0.046	1.244	1.08E-14	Up-Regulated
IVL	1.121	0.114	0.027	0.201	0.010	1.892	2.09E-37	Up-Regulated
L1CAM	1.139	0.130	0.042	0.219	0.004	1.748	3.97E-28	Up-Regulated
MMP1	1.086	0.083	0.005	0.161	0.038	2.805	1.13E-48	Up-Regulated
PSCA	1.081	0.078	0.001	0.155	0.048	1.195	1.18E-10	Up-Regulated
SHCBP1	1.234	0.211	0.018	0.404	0.032	1.031	8.02E-40	Up-Regulated
SPDYC	1.079	0.076	0.002	0.149	0.043	1.116	6.83E-09	Up-Regulated
TMEM105	1.146	0.136	0.015	0.258	0.028	1.159	3.2E-21	Up-Regulated
TMEM45A	1.194	0.177	0.021	0.333	0.026	1.422	6.42E-56	Up-Regulated
TPRXL	1.169	0.156	0.056	0.257	0.002	1.281	3.01E-22	Up-Regulated
WIT1	1.115	0.109	0.005	0.213	0.041	1.346	1.12E-22	Up-Regulated
WT1	1.099	0.095	0.009	0.180	0.030	1.568	1.58E-19	Up-Regulated
ABCA10	0.863	-0.147	-0.280	-0.013	0.031	-1.274	4.3E-28	Down-Regulated
AGBL2	0.809	-0.212	-0.369	-0.055	0.008	-1.273	4.25E-44	Down-Regulated
BTG2	0.791	-0.234	-0.411	-0.058	0.009	-1.391	8.31E-59	Down-Regulated
C1orf168	0.894	-0.113	-0.205	-0.020	0.017	-1.944	4.77E-36	Down-Regulated
C1orf226	0.782	-0.246	-0.394	-0.098	0.001	-1.147	1.62E-31	Down-Regulated
CCDC160	0.899	-0.107	-0.212	-0.002	0.046	-1.148	2.45E-19	Down-Regulated
CCDC74A	0.892	-0.114	-0.223	-0.005	0.040	-1.581	1.34E-34	Down-Regulated
CCDC74B	0.892	-0.115	-0.214	-0.015	0.024	-1.585	5.54E-35	Down-Regulated
CCL19	0.902	-0.103	-0.173	-0.032	0.004	-1.083	4.68E-08	Down-Regulated
CD22	0.866	-0.143	-0.274	-0.013	0.031	-1.241	5.65E-31	Down-Regulated
CLDN19	0.846	-0.167	-0.282	-0.053	0.004	-1.125	5.27E-16	Down-Regulated
CLGN	0.923	-0.080	-0.160	0.000	0.049	-1.601	7.15E-22	Down-Regulated
CLIC6	0.902	-0.103	-0.175	-0.032	0.005	-1.131	1.19E-07	Down-Regulated
COL17A1	0.874	-0.134	-0.205	-0.064	0.000	-1.061	1.62E-07	Down-Regulated
CRIP1	0.881	-0.126	-0.240	-0.013	0.029	-1.222	1.34E-23	Down-Regulated
CYP4F11	0.904	-0.101	-0.180	-0.022	0.012	-1.267	2.76E-13	Down-Regulated
DARC	0.920	-0.083	-0.160	-0.006	0.034	-2.072	1.05E-31	Down-Regulated
EGOT	0.816	-0.204	-0.329	-0.078	0.001	-1.156	5.41E-22	Down-Regulated
ELOVL2	0.911	-0.094	-0.167	-0.020	0.013	-2.433	1.27E-37	Down-Regulated
EVL	0.778	-0.251	-0.430	-0.072	0.006	-1.278	1.83E-53	Down-Regulated
F2RL2	0.893	-0.113	-0.213	-0.013	0.027	-1.621	8.61E-34	Down-Regulated
FGD3	0.865	-0.145	-0.276	-0.015	0.029	-1.308	2.92E-32	Down-Regulated
FLT3	0.910	-0.094	-0.182	-0.007	0.035	-1.766	3.66E-27	Down-Regulated
FREM1	0.840	-0.174	-0.281	-0.067	0.001	-1.070	4.51E-16	Down-Regulated
IL33	0.883	-0.125	-0.217	-0.032	0.008	-1.272	2.79E-18	Down-Regulated
LAMA3	0.878	-0.130	-0.228	-0.032	0.010	-1.040	2.32E-15	Down-Regulated
LOC100128977	0.889	-0.118	-0.217	-0.018	0.020	-1.837	6.35E-34	Down-Regulated
LOC100130148	0.891	-0.116	-0.218	-0.013	0.027	-2.011	1.06E-42	Down-Regulated
LRP1B	0.898	-0.108	-0.200	-0.015	0.022	-1.043	3.52E-07	Down-Regulated
LRRC48	0.830	-0.187	-0.320	-0.054	0.006	-1.670	2.31E-55	Down-Regulated
MEOX1	0.864	-0.146	-0.244	-0.048	0.003	-1.182	2.01E-17	Down-Regulated
MMEL1	0.874	-0.134	-0.259	-0.009	0.036	-1.071	6.68E-21	Down-Regulated
MYO18B	0.885	-0.122	-0.242	-0.002	0.046	-1.427	1.25E-28	Down-Regulated
NKAIN1	0.926	-0.076	-0.152	-0.001	0.047	-1.974	2.92E-25	Down-Regulated
NOS1AP	0.846	-0.167	-0.302	-0.032	0.015	-1.299	5.63E-31	Down-Regulated
NPY1R	0.927	-0.076	-0.139	-0.013	0.018	-2.257	4.91E-22	Down-Regulated
PLD4	0.876	-0.133	-0.259	-0.006	0.040	-1.541	2.63E-40	Down-Regulated
PRICKLE4	0.844	-0.170	-0.336	-0.003	0.045	-1.152	3.02E-34	Down-Regulated
RGL3	0.870	-0.139	-0.234	-0.044	0.004	-1.363	3.68E-24	Down-Regulated
SCUBE2	0.932	-0.070	-0.139	-0.001	0.047	-3.188	8.02E-61	Down-Regulated
SEC14L2	0.875	-0.134	-0.259	-0.009	0.035	-1.125	5.29E-21	Down-Regulated
SEMA3B	0.883	-0.124	-0.228	-0.020	0.019	-1.309	1.57E-22	Down-Regulated
SEMA3G	0.819	-0.199	-0.364	-0.035	0.017	-1.267	3.12E-48	Down-Regulated
SLC27A2	0.906	-0.099	-0.189	-0.008	0.032	-1.423	1.06E-19	Down-Regulated
SLC6A4	0.912	-0.092	-0.171	-0.014	0.021	-1.812	8.02E-17	Down-Regulated
SLC7A3	0.852	-0.160	-0.285	-0.035	0.012	-1.033	7.09E-16	Down-Regulated
SLC7A4	0.917	-0.086	-0.164	-0.009	0.029	-1.598	1.01E-15	Down-Regulated
SNTN	0.878	-0.130	-0.260	0.000	0.049	-1.172	4.76E-23	Down-Regulated
SUSD3	0.865	-0.145	-0.241	-0.048	0.003	-1.985	9.43E-41	Down-Regulated
THSD7B	0.870	-0.140	-0.264	-0.015	0.028	-1.175	9.41E-24	Down-Regulated
TNN	0.863	-0.147	-0.244	-0.050	0.003	-1.317	4.63E-20	Down-Regulated
TPRG1	0.907	-0.097	-0.177	-0.018	0.017	-2.598	1.69E-46	Down-Regulated

*Univariable Cox regression; **Limma R package.

The expression levels of the 13 powerful risk markers in high-risk and low-risk group were visualized in the vioplot and heatmap (**Figures 4D, G**). The results showed that there were significant differences associated with risk score in terms of cluster (P<0.001), PR (P<0.001), ER(P<0.001), HER2(P<0.01) and fustat (P<0.001). The risk score was much higher in cluster1, ER-, PR-, HER2+ and TNBC patients (**Figures 4H–L**). Then, univariate and multivariate Cox regression analysis were performed to test whether the risk signature could be set as independent prognostic factor. As a result, the T stage, N stage, M stage, Stage and risk score were associated with OS by univariate analysis, and only M stage and risk score were still significantly related to OS in multivariate Cox regression analysis (**Figures 4M, N**).

According to our selected risk signature and LASSO Cox regression formula, we calculated risk score and validated our model among 1,356 breast tumor samples in METABRIC dataset. These patients were divided into high risk (n=985) and low risk (n=371) groups by the optimal cut-off point which was obtained by X-tile software. Significantly, Consistent with results of TCGA BRCA tumor samples, the high-risk group also had significantly worse prognosis compared with low-risk group in METABRIC data (**Figure S2A**). AND the AUC value of 0.719 also demonstrated a well-prediction ability (**Figure S2B**). The risk score was much higher in ER-, PR-, HER2+ and advanced pathological grade patients (**Figure S2C–H**). Then, the results of univariate and multivariate Cox regression analysis showed that risk score still could act as an independent prognostic factor among METABRIC patients (**Figures S2I, J**).

### Identification of Hypoxia Associated Differentially Expressed Candidate MicroRNAs

**Figure 5:**
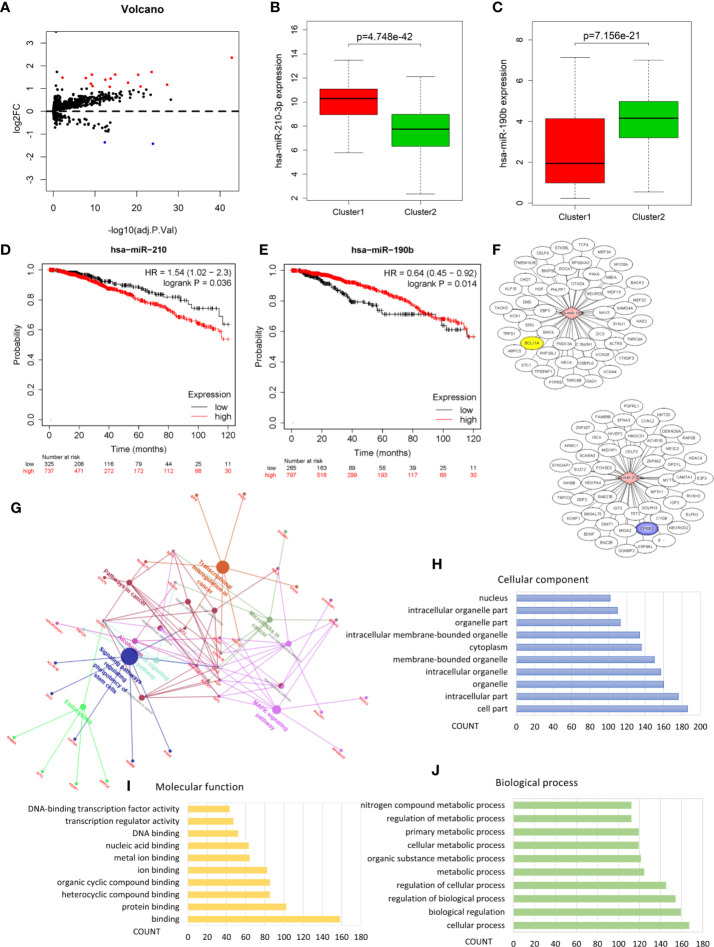
Identification of hypoxia associated differentially expressed candidate microRNAs. **(A)** Volcano plot for differentially expressed microRNAs in cluster1 and cluster2. Red and blue dots represent up-regulated and down-regulated in cluster1 relative to cluster2, respectively (*P* < 0.05, |logFC| >1). **(B, C)** The expression of hsa-miR-210-3p and hsa-miR-190b in cluster1 and cluster2. **(D, E)** The overall survival curves of hsa-miR-210-3p and hsa-miR-190b in TCGA BRCA estimated by the Kaplan Meier plotter. **(F)** The miRNA-mRNA networks for the top50 targets of hsa-miR-210-3p and hsa-miR-190b. **(G)** KEGG pathway enrichment analysis of the target genes of hsa-miR-210-3p and hsa-miR-190b. **(H–J)** The top 10 items of GO analysis: biological processes, molecular functions and cellular components of the target genes of hsa-miR-210-3p and hsa-miR-190b.

Analysis of the miRNA mature strand expression data of TCGA BRCA yielded 15 up-regulated and 2 down-regulated miRNAs in cluster1 compared to cluster2 (**Figure 5A**, **Table 3**). We examined the prognostic value of these 17 differentially expressed miRNAs via the Kaplan-Meier Plotter database. There were 7 up-regulated and only 1 down-regulated miRNAs associated with 120 months’ OS (**Table 3**). We selected one up-regulated and one down-regulated miRNA with the most significant logFC value as the candidate miRNAs. Hsa-miR-210-3p was up-regulated in cluster1, while hsa-miR-190b was down-regulated in cluster1 relative to cluster2 (**Figures 5B, C**). Besides, high expression of hsa-miR-210-3p in BRCA was associated with worse OS (HR=1.54, logrank P=0.036), while high expressed of hsa-miR-190b was related to better OS (HR=0.64, logrank P=0.014) (**Figures 5D, E**).

**Table 3:** Hypoxia associated dysregulated microRNAs in breast cancer.

MicroRNA	logFC	FDR*	regulated	HR	95%CI lower	95%CI upper	logrank P**
hsa-miR-210-3p	2.357	1.51E-43	Up-Regulated	1.54	1.02	2.30	3.60E-02
hsa-miR-224-5p	1.729	2.52E-24	Up-Regulated	1.46	0.98	2.17	6.40E-02
hsa-miR-934	1.614	1.44E-15	Up-Regulated	1.56	1.08	2.26	1.70E-02
hsa-miR-105-5p	1.613	5.53E-10	Up-Regulated	2.26	1.55	3.28	1.20E-05
hsa-miR-135b-5p	1.607	4.47E-21	Up-Regulated	1.29	0.92	1.81	1.50E-01
hsa-miR-1825	1.483	6.51E-03	Up-Regulated	2.08	1.47	2.93	2.50E-05
hsa-miR-767-5p	1.460	1.50E-08	Up-Regulated	1.99	1.37	2.89	2.20E-04
hsa-miR-9-5p	1.436	1.79E-14	Up-Regulated	1.90	1.34	2.69	2.50E-04
hsa-miR-577	1.389	3.37E-13	Up-Regulated	1.30	0.93	1.83	1.30E-01
hsa-miR-452-5p	1.257	1.18E-18	Up-Regulated	1.49	0.97	2.28	6.70E-02
hsa-miR-187-3p	1.214	9.63E-10	Up-Regulated	1.22	0.83	1.78	3.10E-01
hsa-miR-18a-5p	1.169	5.03E-28	Up-Regulated	1.27	0.90	1.79	1.70E-01
hsa-miR-519a-5p	1.115	3.94E-10	Up-Regulated	1.71	1.21	2.43	2.30E-03
hsa-miR-452-3p	1.090	1.19E-21	Up-Regulated	1.49	0.97	2.28	6.70E-02
hsa-miR-455-3p	1.081	2.87E-14	Up-Regulated	1.35	0.96	1.91	8.70E-02
hsa-miR-375	-1.364	4.45E-13	Down-Regulated	1.23	0.88	1.72	2.30E-01
hsa-miR-190b	-1.426	1.29E-24	Down-Regulated	0.64	0.45	0.92	1.40E-02

*Limma R package; **Log-rank P test.

### Pathway Enrichment Analysis Revealed the Role of hsa-miR-210-3p and hsa-miR-190b in Cancer

In order to understand the possible function of hsa-miR-210-3p and hsa-miR-190b in the development of BRCA, KEGG pathway enrichment was utilized to analyze their target genes. Firstly, the target genes of the 2 miRNAs were obtained from the the mirDIP database, an integrative Database of Human microRNA Target Predictions, with the predict score as “very high” (**Table S4** and **Figure 5F**). Then, the pathway enrichment was performed based on these genes, and the results linked these genes to several cancer related pathways: MicroRNAs in cancer, Signaling pathways regulating pluripotency of stem cells, Transcriptional misregulation in cancer, MAPK signaling pathway, Ras signaling pathway, PI3K-Akt signaling pathway, Hepatocellular carcinoma, Pathways in cancer, etc. (**Figure 5G** and **Table S3**). We also performed GO analysis of these target genes including biological processes, molecular functions and cellular components, and the results were shown as Bar graphs in **Figures 5H–J** (more details were shown in **Table S5**).

### Identification of Candidate DEGs Regulated by Candidate MicroRNAs Under Hypoxia Status

**Figure 6:**
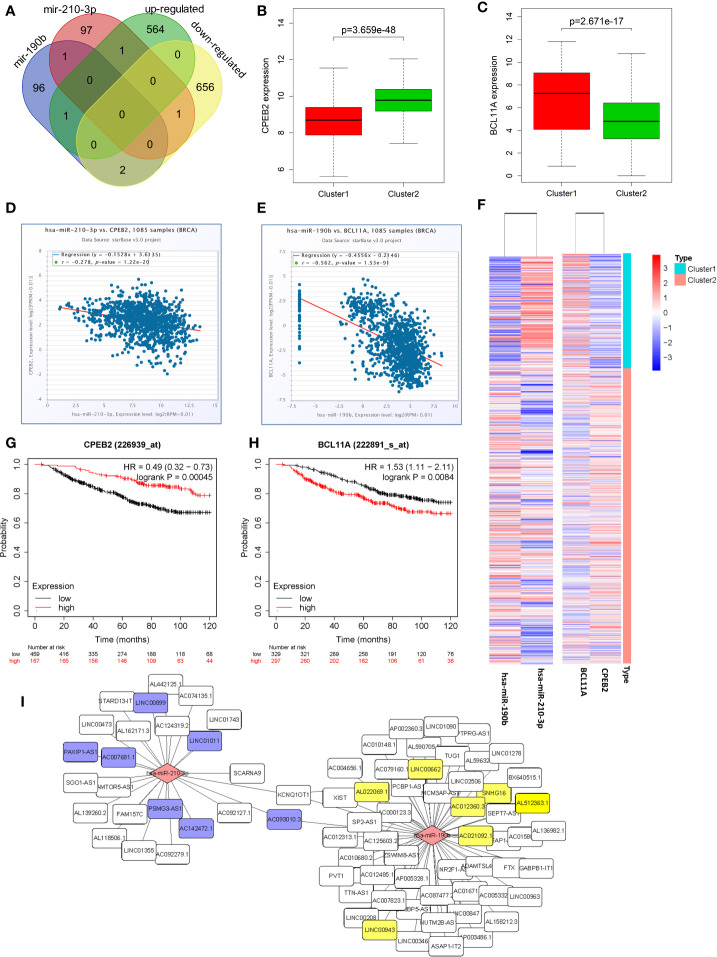
Identification of candidate genes and construction of a ceRNA network. **(A)** Venn diagrams showing the intersection between predicted target genes of hsa-miR-210-3p/hsa-miR-190b and DEGs. **(B, C)** The expression of candidate genes, CPEB2 and BCL11A, in cluster1 and cluster2. **(D, E)** The negative correlations between hsa-miR-210-3p and CPEB2, and between hsa-miR-190b and BCL11A. **(F)** The heatmap of hsa-miR-210-3p, hsa-miR-190b, CPEB2 and BCL11A in cluster1 and cluster2. **(G, H)** The overall survival curves of CPEB2 and BCL11A in breast cancer patients estimated by the Kaplan Meier plotter. **(I)** The miRNA-lncRNA networks of the target lncRNAs of hsa-miR-210-3p and hsa-miR-190b.

Venn diagrams were used to identify the intersection between top100 hsa-miR-210-3p targets and down-regulated genes, as well as between top100 hsa-miR-190b targets and up-regulated genes in cluster1 relative to cluster2 (**Figure 6A**). Among these genes, two candidate DEGs, CPEB2 and BCL11A, were selected. Expression levels were low and high for CPEB2 and BCL11A in cluster1, respectively, and might act as a tumor suppressor gene and a putative oncogene (**Figures 6B, C, F**).

Besides, there were negative correlations between hsa-miR-210-3p and CPEB2 (r=−0.278, P<0.05), and between hsa-miR-190b and BCL11A (r=−0.562, P<0.05) (**Figures 6D, E**). The expression of the two candidate DEGs was associated with the survival of BRCA patients. High expression of CPEB2 was related to better OS (HR=0.49, logrank P<0.05), whereas high expression of BCL11A was associated with worse OS (HR=1.53, logrank P<0.05) (**Figures 6G, H**).

### A Hypoxia Related Competitive Endogenous RNA (ceRNA) Regulation Network

**Figure 7:**
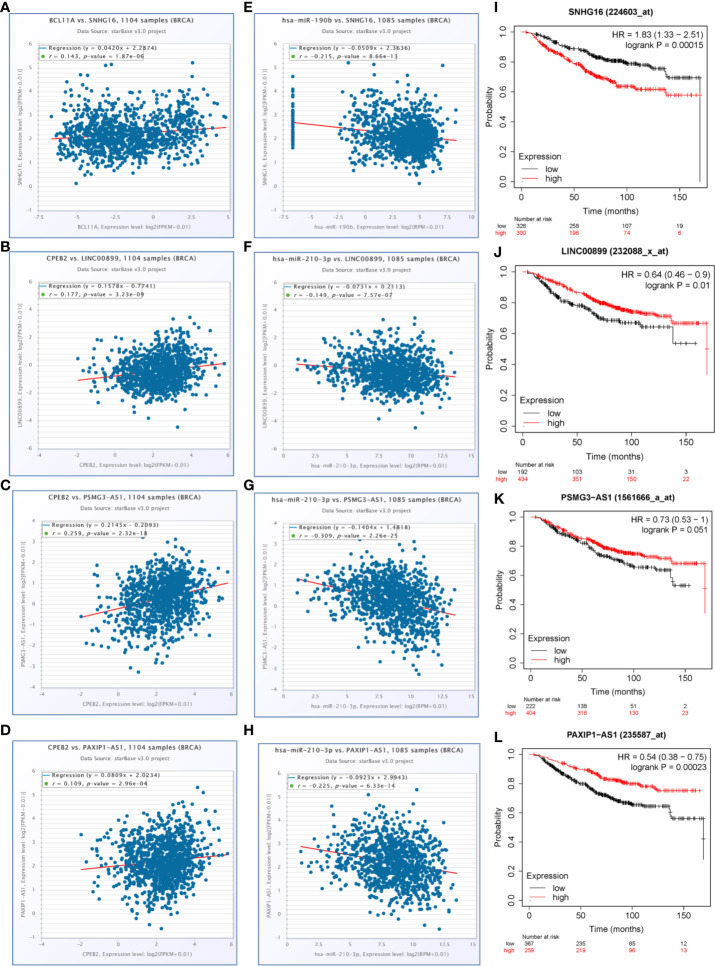
LncRNAs selected for the hypoxia related competitive endogenous RNA (ceRNA) regulation network. **(A–D)** The positive correlations between candidate genes and selected lncRNAs (P<0.05, r>0.1). **(E–H)** The negative correlations between candidate miRNAs and selected lncRNAs (P<0.05, r<−0.1). **(I–L)** The overall survival curves of selected lncRNAs in breast cancer patients estimated by the Kaplan Meier plotter.

**Figure 8:**
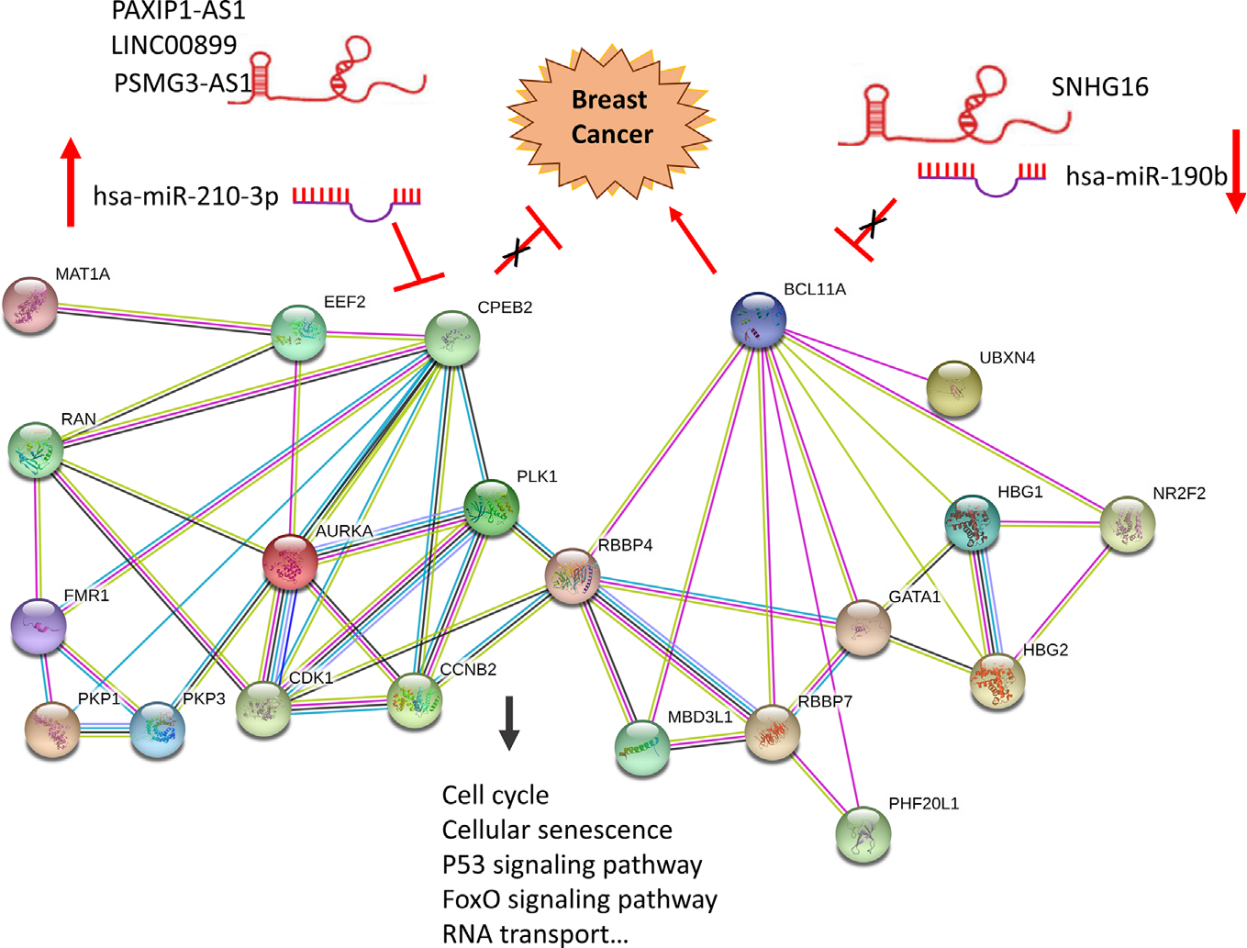
Construction of a hypoxia related ceRNA regulation network based on differentially expressed miRNAs and DEGs. Loss of LINC00899, PSMG3-AS1 and PAXIP1-AS1 leads to increased hsa-miR-210-3p. When overexpressed, hsa-miR-210-3p impedes translation of CPEB2, a tumor suppressor gene in breast cancer under hypoxia status. High expressed SNHG16 can suppress hsa-miR-190b, which leads to increased expression of BCL11A, an oncogene in breast cancer.

To determine whether any lncRNAs might be involved in dysregulated expression of candidate DEGs and miRNAs, we firstly used the Starbase database to predict the target lncRNAs of hsa-miR-210-3p and hsa-miR-190b (**Table S4** and **Figure 6I**). Then we chose the lncRNAs which are not only negatively related to candidate miRNAs but also positively related to CPEB2 and BCL11A based on ceRNA theory (**Table 4**). Among the selected lncRNAs, 4 of them, SNHG16, LINC00899, PSMG3-AS1 and PAXIP-AS1, were significantly associated with survival (**Figure 7**). High expression of SNHG16 could predict a poor prognosis, while high expression of LINC00899, PSMG3-AS1 and PAXIP-AS1 could predict a better prognosis. We constructed a local protein network between the proteins CPEB2 and BCL11A, we then added candidate miRNAs and the 4 lncRNAs to this network. In this network, loss of LINC00899, PSMG3-AS1 and PAXIP1-AS1 leads to increased hsamiR-210-3p. When overexpressed, hsa-miR-210-3p impedes translation of CPEB2, a tumor suppressor gene in breast cancer under hypoxia status. High expressed SNHG16 can suppress hsa-miR-190b, which leads to increased expression of BCL11A, an oncogene in breast cancer (**Figure 8**). Thus, this dysregulated ceRNA network can result in progression of breast cancer under hypoxia situation.

**Table 4:** The correlation coefficient of lncRNA-microRNA and lncRNA-mRNA in TCGA BRCA.

LncRNA	MicroRNA	Correlation coefficient	mRNA	Correlation coefficient
AC093010.3	hsa-miR-210-3p	−0.382	CPEB2	0.050
LINC01011	hsa-miR-210-3p	−0.176	CPEB2	0.140
AC142472.1	hsa-miR-210-3p	−0.203	CPEB2	−0.036
PSMG3-AS1	hsa-miR-210-3p	−0.309	CPEB2	0.259
AC007681.1	hsa-miR-210-3p	−0.113	CPEB2	0.103
LINC00899	hsa-miR-210-3p	−0.149	CPEB2	0.177
PAXIP1-AS1	hsa-miR-210-3p	−0.225	CPEB2	0.109
SNHG16	hsa-miR-190b	−0.215	BCL11A	0.143
AC021092.1	hsa-miR-190b	−0.185	BCL11A	0.143
LINC00662	hsa-miR-190b	−0.135	BCL11A	0.181
LINC00943	hsa-miR-190b	−0.313	BCL11A	0.442
AC012360.3	hsa-miR-190b	−0.110	BCL11A	0.135
AL512363.1	hsa-miR-190b	−0.263	BCL11A	0.289
AL022069.1	hsa-miR-190b	−0.116	BCL11A	0.185

## Discussion

The complete tumor tissue includes not only cancer cells, but also the surrounding vessels, lymphatic vessels, fibroblasts, inflammatory cells, and extracellular matrix. It also includes a variety of interstitial cells and biomolecules infiltrating them. These are collectively referred to as the tumor microenvironment (34, 35). The abnormal vascular network in solid tumors and the excessive oxygen demand of rapidly growing cancer cells lead to hypoxia in tumor tissues. Hypoxic and acidic microenvironment is one of the most important components of tumor microenvironment. Cancer cells adapt to and rely on these microenvironments, leading to the diversity and instability of gene mutations, activating multiple signaling pathways and survival factors, which contribute to angiogenesis, metabolic reprogramming, epithelial–mesenchymal transition, invasion, metastasis, cancer stem cell maintenance, immune evasion, and resistance to chemotherapy and radiation therapy (5, 36). Therefore, understanding the effect of hypoxia on molecular mechanism is essential to improve the outcome of cancer treatment.

In our current study, we selected 13 hypoxia related gene signature which can well demonstrate the hypoxia status based on published studies. These 13 genes make up a common hypoxia signature which will be up-regulated and are consistently co-expressed with previously validated hypoxia-regulated genes under hypoxic conditions in various cancers. They are a small number of top-ranked genes with the highest connectivity and the most prognostic in hypoxia co-expression cancer networks, including head and neck, breast and lung cancers (12). And according to their expression, we defined the hypoxia status of breast cancer tissues to divide these breast cancer samples into two groups, namely cluster1 and cluster2. Considering that the expression of the 13 genes in cluster1 is higher than that of cluster2, we defined cluster1 as the “hypoxic subgroup”. For the association between hypoxia status and clinicopathological characteristics, there were more ER-, PR-, Her2+ and triple negative breast cancer patients in cluster1 than cluster2. This result indicates that the hypoxic state is closely related to the malignant phenotype of breast cancer.

An increasing number of studies indicate that the hypoxia status of tumor tissue is an important reason for promoting tumor immunosuppression and resistance to immunotherapy. Tumor hypoxic regions can recruit immunosuppressive cells such as myeloid derived suppressor cells (MDSC), tumor-associated macrophage (TAM) and Tregs, and can inhibit the activation of CD8+T cells and CD4+T cells (33, 37). Hypoxia can increase the expression and secretion of CCL28 in ovarian cancer cells. CCL28 is an inducer of Tregs and has an immunosuppressive function on CD8+ T cells (38). In the presence of hypoxia and TGF-β, CD4+ T cells upregulated Foxp3 through the binding of HIF-1 to the promoter region of Foxp3, which induced the differentiation of Tregs and enhances immunosuppression (39). Therefore, we compared the infiltration of 24 immune cell types including 18 T-cell subtypes and 6 other immune cells in cluster1 and cluster2. The results showed that, compared with cluster 2, the infiltration of CD8 + T cells and CD4 + T cells in cluster 1 was lower, while the infiltration of nTreg cells and iTreg cells in cluster 1 was higher. This result indicates that there is an immunosuppressive state in cluster1. In addition, this result in turn confirmed that we initially defined cluster1 as the “hypoxic subgroup” was correct. The recent research results of Shaoquan Zheng et al. are in strong agreement with ours. They constructed a combined hypoxia and immune index based on 3 hypoxia-related genes and 7 immune-related genes for triple-negative breast cancer samples in Gene Expression Omnibus (GEO), TCGA and METABRIC by silico analyses, and patients were divided into hypoxia^high^/immune^low^ and hypoxia^low^/immune^high^ groups. The key markers of hypoxia (ALDOA, ENO1, LDHA, etc.) are highly expressed in the hypoxia^high^/immune^low^ group, while a higher percentage of CD8+ T cells was observed in the hypoxia^low^/immune^high^ group (40). In this case, both their and our results confirmed that there is a strong positive correlation between the hypoxia status of tumor and immunosuppression.

The response to hypoxia includes a series of adaptation mechanisms that promote tumor cells survival (41). Basel Abu-Jamous et al. jointly analyzed 16 heterogeneous breast cancer cell lines transcriptome datasets under hypoxia-related conditions and identified a series of genes that were up-regulated under hypoxia, such as BRAF, EGLN1, EGLN3, GAB1, MAP2K1, MET, SLC2A1, VEGFA, and VEGFC. They are well described as part of the hypoxic transcriptome and are HIF targets involved in the response to hypoxia, positive regulation of the I-kappaB kinase/NF-kappaB cascade, carbohydrate metabolism, glycolysis and other pathways and GO functions (42). Further, we analyzed the differentially expressed genes of cluster1 and cluster2 to explore the molecular mechanism of breast cancer tissue changes under hypoxia. Among them, some important DEGs clustered significantly into pathways related to hypoxia and tumor invasion and metastasis, such as HIF-1 signaling pathway, Transcriptional misregulation in cancer, Bladder cancer, Central carbon metabolism in cancer, Glycolysis/Gluconeogenesis, AMPK signaling pathway, etc. Similarly, these results in turn confirmed that our hypoxic classification of tumor samples was correct. The HIF-1 signaling and Glycolysis/Gluconeogenesis pathways play a vital role in promoting the occurrence and development of tumors under hypoxic conditions. As is reported, cancer cells can use both conventional oxidative metabolism and glycolytic anaerobic metabolism. However, even in the presence of oxygen, their proliferation is also characterized by increased glycolytic metabolism which is called Warburg effect. HIF 1 as a major hypoxia-inducible transcription factor can promote the dissociation between glycolysis and tricarboxylic acid cycle. This process limits the effective production of ATP and citric acid which would otherwise prevent glycolysis. The Warburg effect is also conducive to alkaline pH in tumor cells, which drives cancer cell proliferation (enhancing cell cycle progression and glycolysis) and cancer aggressiveness (resistance to immune response, cytotoxic drugs and apoptosis). This effect even leads to epigenetic and genetic changes which cause cells to appear a variety of new phenotypes, thereby enhancing the growth and aggressiveness of cancer cells (43). Therefore, our results mean that these DEGs between cluster1 and cluster2 may play a role in promoting tumor progression through their functions according to existing research results.

Enriched studies indicate that hypoxia-related gene signatures generated in vitro and in vivo have prognostic power in breast cancer and other cancers. Inna Y. Gong et al. explored several datasets from GEO database based on four published hypoxia signatures [Buffa (12), Winter (44), Hu (45), and Sorensen (46)], and confirmed to a certain extent that hypoxia-related gene signatures had potential to be used as biomarkers to predict survival of early breast cancer (47). By contrast, Maud H W Starmans et al. identified 295 up-regulated and 164 down-regulated genes under hypoxia in breast (MCF7), colon (HT29) and prostate (DU145) carcinoma cells in vitro, but they found that none of these in vitro derived signatures consisting of hypoxia-induced genes are prognostic when in a much larger cohort of breast cancer patients in vivo (48). In an effort to bolster clinical tools for hypoxic understanding of breast cancer, we also developed a prognostic signature associated with hypoxia. By univariate Cox and LASSO Cox regression analysis, we constructed a new risk signature which was not reported before based on 3 up-regulated and 9 down-regulated genes in cluster1. Besides, our 12-gene signature showed a well-prediction ability to provide new perspectives for the identification of breast cancer with a high risk of death. And the risk score is much higher in cluster1, ER-, PR-, HER2+, TNBC, and advanced Grade patients, which indicates that the increased risk score also predicts a malignant breast cancer molecular phenotype. Some studies have confirmed the role and function of genes in this risk signature. For example, CCL19 inhibited cell proliferation, migration, and invasion in gastric cancer by activating the CCR7/AIM2 signaling pathway, which could be a potential therapeutic approach (49). EGOT reduced the vitality and migration of breast cancer cells by inactivating the Hedgehog pathway (50). COL17A1 as a target of p53 can also inhibit the migration and invasion of breast cancer cells (51).

MiRNAs and lncRNAs are identified as key regulators of gene expression in various biological and pathological processes, including cancer (52). Further we use data contained in databases such as StarBase, mirDIP, Kaplan-Meier Plotter and TCGA, based on ceRNA theory, we identified potential ncRNA regulatory pathways involving a tumor suppressor and an oncogene, LINC00899/PSMG3-AS1/PAXIP1-AS1- hsa-miR-210-3p-CPEB2 and SNHG16- hsa-miR-190b-BCL11A ceRNA regulation networks, and built a local PPI network which might promote the development of breast cancer under hypoxia. Experimental results are consistent with some of our predictions. It is reported that miR-210 was upregulated by HIF-1α in the stromal cells of giant cell tumors of bone (53). Downregulation of miR-210 inhibited growth of tumors, such as glioblastoma and osteosarcoma (54, 55). In addition, CPEB2 has been shown to act as a tumor suppressor gene in breast cancer. In MCF7 cells, CPEB2 gene knockdown mediated by siRNA promotes carcinogenic properties in vitro, promotes EMT, migration, invasion, proliferation and stem cell-like phenotype of cells (56). Breast cancer-derived exosomes induced CD73 + γδ1 Treg cells by transmitting lncRNA SNHG16, while CD73 + γδ1 Treg cells exert an immunosuppressive effect through adenosine production (57). Besides, by directly interacting with the 3’UTR of Bcl2, miR-190b induces osteosarcoma cell apoptosis and confers radio-sensitivity to gastric cancer cells (58, 59). According to reports, BCL11A acts as a carcinogenic gene for a variety of human cancers, such as breast cancer (60), laryngeal squamous cell carcinoma (61), high-risk neuroblastoma (62), non-small cell lung cancer (63) etc. In addition, the expression of PSMG3-AS1 in breast cancer tumor tissues and cell lines was increased, and PSMG3-AS1 as a sponge of miR-143-3p enhanced the proliferation and migration ability in the pathogenesis of breast cancer (64).

In conclusion, our research has provided an understanding of potential carcinogenesis mechanism and molecular prognostic markers of breast cancer under hypoxic conditions from multiple levels by in silico analyses. We hope that our research can provide a new theoretical basis for exploring the carcinogenic and progression mechanisms of breast cancer. However, it is undeniable that our research still has some limitations. The data from the TCGA database does not directly provide values for hypoxia status, for example, O_2_ levels. At the same time, we have only analyzed and constructed relevant ceRNA regulatory networks for hsa-miR-190b and hsa-miR-210-3p, but not other miRNAs. The next important step is to use functional experiments to verify our predictions.

## Data Availability Statement

The original contributions presented in the study are included in the article/**Supplementary Material**. Further inquiries can be directed to the corresponding author.

## Author Contributions

P-JG and J-WZ contributed to the conception of the study. P-JG, Y-CS, and S-RH contributed to experimental technology and experimental design. P-JG, S-RH, and Y-FZ performed the data analyses. P-JG, X-NY, J-JX, and W-NY wrote the manuscript. LW and J-WZ supervised the study. All authors contributed to the article and approved the submitted version.

## Funding

This work was supported by funds from Health commission of Hubei Province scientific research project (WJ2019H020, WJ2019H028).

## Conflict of Interest

The authors declare that the research was conducted in the absence of any commercial or financial relationships that could be construed as a potential conflict of interest.

## Supplementary Material

Supplementary Table 1 Cellular pathways of 13 hypoxia related gene signature.

Supplementary Table 2 Pathway and GO enrichment results of the sub-network DEGs.

Supplementary Table 3 Cellular pathways of 12 genes of risk signature.

Supplementary Table 4 Target mRNAs of hsa-miR-210-3p and hsa-miR-190b.

Supplementary Table 5 Pathway and GO enrichment results of target mRNAs of hsa-miR-210-3p and hsa-miR-190b.

Supplementary Table 6 Target lncRNAs of hsa-miR-210-3p and hsa-miR-190b. Supplementary material for detailed explanation of the Consensus clustering results.

Supplementary Methods Detailed explanation of the Consensus clustering results.

Supplementary Figure 1 Boxplots of 24 immune cell types between cluster 1 and cluster 2.

Supplementary Figure 2 Risk signature tested in METABRIC dataset. **(A)** Kaplan-Meier overall survival (OS) curves for patients in high- and low-risk group. **(B)** ROC analysis and AUC value of the ROC curve for risk score. **(C)** The expression of the 12 powerful prognostic markers in high-risk group and low-risk group (Wilcox Test). **(D)** The heatmap shows the expression of the 12 powerful prognostic markers in high-risk group and low-risk group (Chi-square Test). The distribution of clinicopathological characteristics was compared between the high-risk and low-risk groups. **(E–H)** The relationship between Rick score and ER, PR, HER2, Grade (Wilcox Test). **(I)** Univariate Cox regression analysis of the associated between clinicopathological features (including risk score) and overall survival of patients. **(J)** Multivariate Cox regression analysis of the associated between clinicopathological features (including risk score) and overall survival of patients. **P* < 0.05, ***P*< 0.01, and ****P*< 0.001.

## Abbreviation

TCGA, The Cancer Genome Atlas; BRCA, Breast invasive carcinoma; GO, Gene Ontology; KEGG, Kyoto Encyclopedia of Genes and Genomes; BP, biological processes; CC, cellular components; MF, molecular functions; HR, hazard ratio; CI, Confidence interval. ER, estrogen receptor; PR, progesterone receptor; HER2, human epidermal growth factor receptor 2; VEGF, Vascular endothelial growth factor; HIF-1α, Hypoxia-inducible factor 1-alpha; CCL28, Chemokine (C-C motif) ligand 28; lncRNA, long non-coding RNA; miRNA, microRNA; circRNA, Circular RNA; ceRNAs, competitive endogenous RNAs; OS, overall survival; DEGs, differentially expressed genes.
