# Corrigendum: Hypoxia-Associated Prognostic Markers and Competing Endogenous RNA Co-Expression Networks in Breast Cancer

**DOI:** 10.3389/fonc.2020.637481

**Published:** 2021-01-29

**Authors:** Peng-Ju Gong, You-Cheng Shao, Si-Rui Huang, Yi-Fan Zeng, Xiao-Ning Yuan, Jing-Jing Xu, Wei-Nan Yin, Lei Wei, Jing-Wei Zhang

**Affiliations:** ^1^ Department of Breast and Thyroid Surgery, Zhongnan Hospital, Hubei Key Laboratory of Tumor Biological Behaviors, Hubei Cancer Clinical Study Center, Wuhan University, Wuhan, China; ^2^ Department of Pathology and Pathophysiology, Hubei Provincial Key Laboratory of Developmentally Originated Disease, School of Basic Medical Sciences, Wuhan University, Wuhan, China

**Keywords:** hypoxia, breast cancer, The Cancer Genome Atlas, ceRNA, prognosis

In the original article, there was a mistake in the legend for **Figure 8** as published.

The correct legend appears below.

In the original article, there was a mistake in **Figure 8** and **Table 4** as published. Due to our carelessness, “hsa-miR-210-3p” and “hsa-miR-190b” were wrongly written as “has-mir-210-3p” and “has-mir-190b”.

The corrected **Figure 8** and **Table 4** appears below.

**Figure 8:**
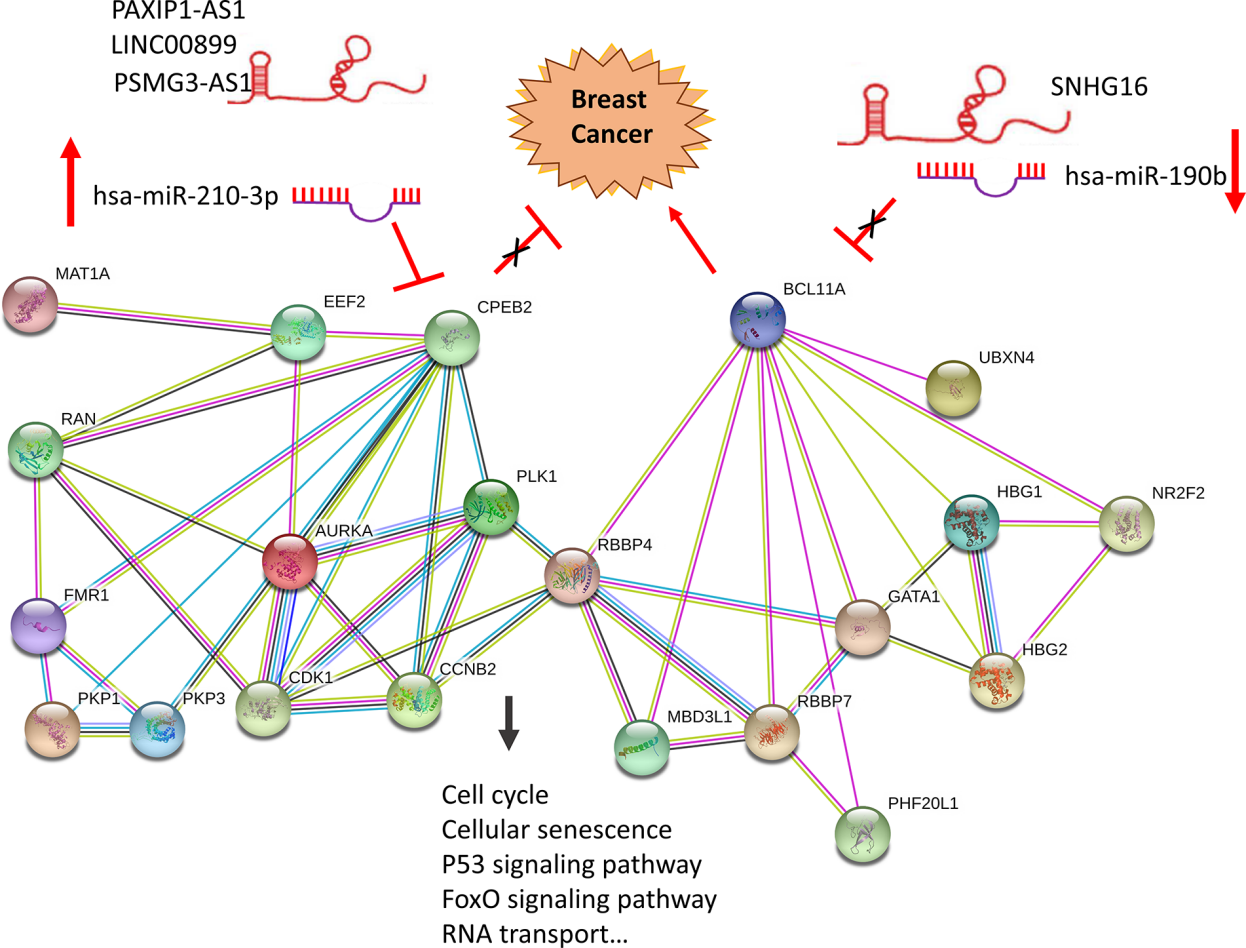
Construction of a hypoxia related ceRNA regulation network based on differentially expressed miRNAs and DEGs. Loss of LINC00899, PSMG3-AS1 and PAXIP1-AS1 leads to increased hsa-miR-210-3p. When overexpressed, hsa-miR-210-3p impedes translation of CPEB2, a tumor suppressor gene in breast cancer under hypoxia status. High expressed SNHG16 can suppress hsa-miR-190b, which leads to increased expression of BCL11A, an oncogene in breast cancer.

**Table 4:** The correlation coefficient of lncRNA-microRNA and lncRNA-mRNA in TCGA BRCA.

LncRNA	MicroRNA	Correlation coefficient	mRNA	Correlation coefficient
AC093010.3	hsa-miR-210-3p	−0.382	CPEB2	0.050
LINC01011	hsa-miR-210-3p	−0.176	CPEB2	0.140
AC142472.1	hsa-miR-210-3p	−0.203	CPEB2	−0.036
PSMG3-AS1	hsa-miR-210-3p	−0.309	CPEB2	0.259
AC007681.1	hsa-miR-210-3p	−0.113	CPEB2	0.103
LINC00899	hsa-miR-210-3p	−0.149	CPEB2	0.177
PAXIP1-AS1	hsa-miR-210-3p	−0.225	CPEB2	0.109
SNHG16	hsa-miR-190b	−0.215	BCL11A	0.143
AC021092.1	hsa-miR-190b	−0.185	BCL11A	0.143
LINC00662	hsa-miR-190b	−0.135	BCL11A	0.181
LINC00943	hsa-miR-190b	−0.313	BCL11A	0.442
AC012360.3	hsa-miR-190b	−0.110	BCL11A	0.135
AL512363.1	hsa-miR-190b	−0.263	BCL11A	0.289
AL022069.1	hsa-miR-190b	−0.116	BCL11A	0.185

In the original article, there was an error. “LINC00899/PSMG3-AS1/PAXIP1-AS1- hsa-miR-210-3p-CPEB2 and SNHG16- hsa-miR-190b-BCL11A” were wrongly written as “SNHG16-hsa-miR-210-3p-CPEB2 and LINC00899/PSMG3-AS1/PAXIP-AS1-hsa-miR-190b-BCL11A”.

A correction has been made to the *Abstract*, under ‘*Results*’:

“Predictions based on the LINC00899/PSMG3-AS1/PAXIP1-AS1- hsa-miR-210-3p-CPEB2 and SNHG16-hsa-miR-190b-BCL11A ceRNA regulation networks indicated that the two genes might act as tumor suppressor and oncogene, respectively.”

A correction has also been made to *Discussion*, paragraph 6:

“Further we use data contained in databases such as StarBase, mirDIP, Kaplan-Meier Plotter and TCGA, based on ceRNA theory, we identified potential ncRNA regulatory pathways involving a tumor suppressor and an oncogene, LINC00899/PSMG3-AS1/PAXIP1-AS1- hsa-miR-210-3p-CPEB2 and SNHG16- hsa-miR-190b-BCL11A ceRNA regulation networks, and built a local PPI network which might promote the development of breast cancer under hypoxia.”

In the original article, there was another error. “SNHG16” and “LINC00899, PSMG3-AS1 and PAXIP-AS1” were incorrectly interchanged.

A correction has been made to Results, under *‘A Hypoxia Related Competitive Endogenous RNA (ceRNA) Regulation Network’*:

“In this network, loss of LINC00899, PSMG3-AS1 and PAXIP1-AS1 leads to increased hsa-miR-210-3p. When overexpressed, hsa-miR-210-3p impedes translation of CPEB2, a tumor suppressor gene in breast cancer under hypoxia status. High expressed SNHG16 can suppress hsa-miR-190b, which leads to increased expression of BCL11A, an oncogene in breast cancer.”

In the original article, there was a further error. The authors stated that CPEB2 and SNHG16 have been shown to act as tumor suppressor genes in breast cancer.

A correction has been made to *Discussion*, paragraph 6:

“In addition, CPEB2 has been shown to act as a tumor suppressor gene in breast cancer.”

The authors apologize for these errors and state that they do not change the scientific conclusions of the article in any way.

The original article has been updated.

