# Supplementary material for: Hypoxia-Associated Prognostic Markers and Competing Endogenous RNA Co-Expression Networks in Breast Cancer
Source: Front Oncol. 2020 Dec 2;10:579868. doi: 10.3389/fonc.2020.579868 (PMC7738636; doi:10.3389/fonc.2020.579868)
Supplement: Supplementary Methods — Detailed explanation of the Consensus clustering results. [file DataSheet_1.docx]

Consensus clustering is a method that provides quantitative evidence to determine the number and members of possible clusters in a data set, such as microarray gene expression. The Consensus Clustering method involves subsampling from a set of items, such as microarrays, and determines clusterings of specified cluster counts (*K*).Then, pairwise consensus values, the proportion that two items occupied the same cluster out of the number of times they occurred in the same sub sample, are calculated and stored in a symmetrical consensus matrix for each *K.* The consensus matrix is summarized in several graphical displays that enable a user to decide upon a reasonable cluster number and membership.

In the CDF curve of a consensus matrix, the lower left portion represents sample pairs rarely clustered together, the upper right portion represents those almost always clustered together, whereas the middle segment represents those with ambiguous assignments in different clustering runs. The proportion of ambiguous clustering (PAC) score measure quantifies this middle segment; and is defined as the fraction of sample pairs with consensus indices falling in the interval (u1, u2) ∈ [0, 1] where u1 is a value close to 0 and u2 is a value close to 1 (for instance u1=0.1 and u2=0.9). A low value of PAC indicates a flat middle segment, and a low rate of discordant assignments across permuted clustering runs. One can therefore infer the optimal number of clusters by the *K* value having the lowest PAC. Based on this theory, *K*=2 should be selected for the optimal number of clusters in our study (Figure 1 E).


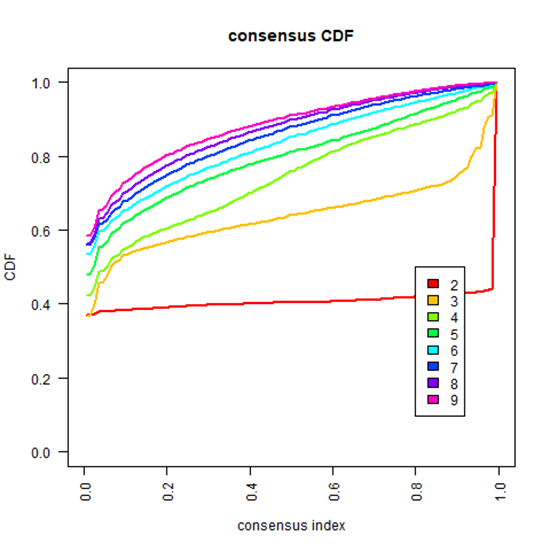


U2

U1

When K=2, PAC could reach the lowest value. (PAC for each K is CDF_K_(U2)-CDF_K_(U1), and optimal K is the K with the lowest PAC value)

This tracking plots shows the cluster assignment of items (columns) for each *K* (rows) by color. The colors correspond to the colors of the consensus matrix class asssignments. Hatch marks below the plot indicate items/samples. This plot provides a view of item cluster membership across different *K*. Items that change clusters often (changing colors within a column) are indicative of unstable membership. Clusters with an abundance of unstable members suggest an unstable cluster. The consensus matrices are ordered by the consensus clustering which is depicted as a dendrogram atop the heatmap. To aid analysis, the cluster memberships are marked by colored rectangles between the dendrogram and heatmap according to a legend within the graphic.


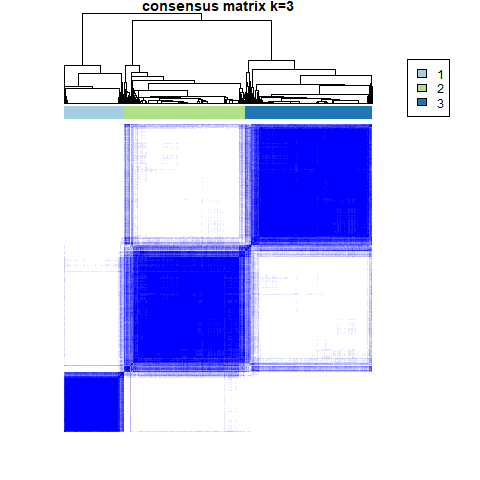

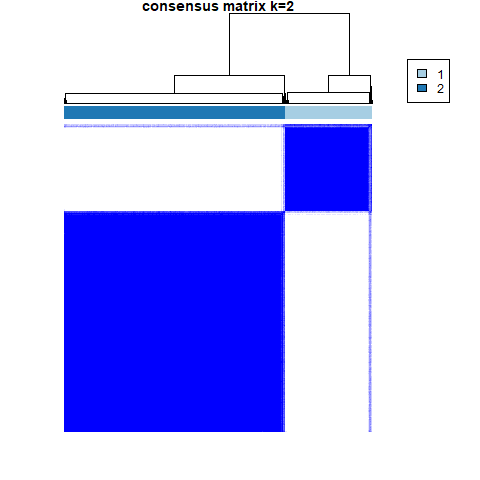

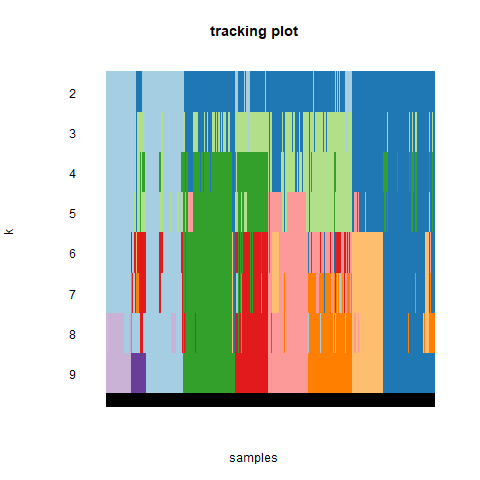


The interference between subgroups could be reduced to minimal when K=2 was selected for consensus clustering analysis (Figure 1G, H, I).

Based on these reasons, *K*=2 is the optimal value for cluster. Thus, two subgroups named cluster1 and cluster2 were identified.
